# Nutrition and Hydration Strategies for Heatwave Adaptation: A Narrative Review

**DOI:** 10.3390/nu18152502

**Published:** 2026-08-03

**Authors:** Stefania D’Angelo

**Affiliations:** Department of Medical, Human Movement, and Well-Being Sciences (DiSMMeB), University of Naples Parthenope, 80133 Naples, Italy; stefania.dangelo@uniparthenope.it

**Keywords:** climate change, heatwaves, extreme heat, thermoregulation, hydration, dehydration, electrolyte balance, nutrition, Mediterranean diet, vulnerable populations, heat-health action plans, public health nutrition

## Abstract

Climate change is increasing the frequency, intensity, and duration of heatwaves, making extreme heat a recurrent public health challenge with important nutritional implications. High ambient temperatures affect human health through thermoregulatory strain, sweating, dehydration, electrolyte imbalance, cardiovascular and renal stress, impaired cognitive and physical performance, and exacerbation of chronic diseases. Diet and hydration may therefore represent modifiable, although still underinvestigated, components of heat-health prevention. This narrative review synthesizes evidence on hydration, electrolyte balance, meal composition, dietary quality, vulnerable populations, Mediterranean dietary patterns, seasonality, environmental sustainability, and public health strategies for reducing heat-related health risks. Evidence from climate-health research, physiology, nutrition science, occupational and sports medicine, geriatrics, pediatrics, and public health guidance was integrated. Particular attention was given to distinguishing direct heat-health evidence from recommendations based on indirect evidence, physiological plausibility, or expert consensus. Regular water intake, water-rich foods, avoidance of alcohol, moderation of sugar-sweetened beverages, and individualized electrolyte replacement during prolonged or intense sweating are supported by physiological rationale and public health guidance, although direct heatwave-specific intervention studies remain limited. Dietary recommendations should prioritize smaller, digestible, nutrient-dense meals that preserve nutritional adequacy while reducing unnecessary digestive and metabolic burden. However, evidence on meal size, macronutrient composition, and heat-related outcomes remains largely indirect. Vulnerable groups, including older adults, children, pregnant women, individuals with chronic diseases, outdoor workers, athletes, and socioeconomically disadvantaged populations, require tailored strategies. The Mediterranean diet may provide a useful regional model because it emphasizes seasonal plant foods, fruits and vegetables with high water content, legumes, whole grains, olive oil, culinary simplicity, and environmental sustainability; however, its specific role in heat adaptation requires further study. Integrating nutrition and hydration into heat-health action plans may improve preparedness and support climate-resilient food systems. Future studies should evaluate hydration protocols, meal patterns, Mediterranean diet adherence, biomarkers of nutritional heat resilience, and nutrition-sensitive interventions in real-world heat exposure settings.

## 1. Introduction

Climate change is increasingly recognized as a major determinant of human health, with rising ambient temperatures and more frequent, intense, and prolonged heatwaves being one of its most direct and measurable health threats [1,2]. Extreme heat is no longer an episodic environmental hazard, but an emerging and recurrent public health challenge, particularly in regions already exposed to high summer temperatures, including the Mediterranean area. Heat exposure is associated with increased morbidity and mortality through cardiovascular, renal, respiratory, metabolic, neurological, and mental health effects, with a disproportionate impact on vulnerable populations [1,2].

The human response to heat exposure depends on complex thermoregulatory mechanisms aimed at maintaining core body temperature within a narrow physiological range. Cutaneous vasodilation and sweating are central adaptive responses, but they also increase water and electrolyte losses, particularly during prolonged exposure, physical activity, occupational heat stress, or insufficient access to fluids and cooling strategies [2,3]. When fluid losses are not adequately replaced, hypohydration and dehydration may occur, impairing cardiovascular stability, renal function, cognitive performance, and thermoregulatory efficiency [2,3,4]. These processes are particularly relevant in older adults, children, pregnant women, individuals with chronic diseases, outdoor workers, athletes, and socioeconomically disadvantaged populations, who may have reduced physiological reserve, altered thirst perception, medication-related vulnerability, or limited access to safe water and cooling environments [1,2,5].

Hydration is therefore a central component of heat-health prevention. The European Food Safety Authority has established dietary reference values for total water intake, including water from beverages and foods, of approximately 2.0 L/day for adult women and 2.5 L/day for adult men under conditions of moderate environmental temperature and physical activity [6]. However, these values may need to be adapted during hot weather, heatwaves, fever, physical exertion, or increased sweating. Public health recommendations from national and international agencies consistently emphasize regular fluid intake, avoidance of alcohol, moderation of sugar-sweetened and caffeinated beverages, and early recognition of dehydration symptoms during periods of high temperature [1,7,8]. Nevertheless, hydration advice is often delivered as a general behavioral recommendation, while its integration with broader dietary strategies, vulnerable-population guidance, and heat-health action plans remains less systematically developed.

Dietary patterns may influence hydration and heat-related vulnerability through several pathways, although the direct evidence remains limited. Foods with high water content, such as fruits and vegetables, contribute to total water intake and provide micronutrients and bioactive compounds that may support overall metabolic health. Conversely, large, high-fat, energy-dense meals may increase postprandial thermogenesis and digestive burden, potentially worsening thermal discomfort during hot weather. Alcohol intake can promote diuresis and impair behavioral responses to heat, whereas excessive consumption of sugar-sweetened beverages may contribute to suboptimal cardiometabolic profiles without providing meaningful protection against heat stress [7,8]. In physically active individuals and outdoor workers, prolonged sweating may also require attention to sodium and other electrolytes, although electrolyte replacement should be contextualized according to duration and intensity of exposure, sweat losses, health status, and the risk of inappropriate supplementation [3,4]. Importantly, several dietary recommendations during heatwaves are based mainly on physiological plausibility, public health guidance, or extrapolation from sports and occupational heat research rather than on direct intervention studies conducted during real-world heatwaves.

Beyond individual physiological needs, nutrition in the context of extreme heat may also be considered within a broader framework of climate adaptation and sustainable food systems. The Mediterranean dietary pattern, characterized by high consumption of vegetables, fruits, legumes, whole grains, nuts, olive oil, and moderate intake of fish and other animal-source foods, has been widely investigated for its health-promoting properties and is increasingly discussed as a model of sustainable eating [9]. In Mediterranean regions, this dietary pattern may provide a useful regional example because it emphasizes seasonal plant foods, water-rich fruits and vegetables, culinary simplicity, and minimally processed foods. However, its specific role in improving heat tolerance, hydration status, or heat-related clinical outcomes has not been directly demonstrated; therefore, it should be interpreted as a culturally rooted and potentially sustainable dietary model rather than as a proven heat-protective intervention.

Despite the relevance of this topic, the evidence specifically addressing dietary strategies during heatwaves remains fragmented. Most available recommendations are derived from physiological studies on heat stress and hydration, sports and occupational medicine, public health guidance, and general principles of healthy and sustainable nutrition [1,3,4,7,8]. Relatively few studies have directly examined how dietary patterns, meal composition, food water content, and electrolyte intake affect heat-related outcomes in the general population or in vulnerable groups. This gap is important because heat-health action plans often prioritize environmental cooling, behavioral advice, and emergency preparedness, while nutrition is rarely integrated as a structured part of preventive strategies [4,5]. A clearer distinction is therefore needed between direct heat-health evidence, evidence extrapolated from athletic or occupational settings, general nutrition evidence, mechanistic plausibility, and public-health consensus.

The aim of this narrative review is to synthesize current evidence on the role of diet and hydration in reducing health risks associated with high ambient temperatures and heatwaves in the context of climate change. Specifically, this review will examine the physiological basis of heat stress and fluid balance, dietary and hydration recommendations for the general population, specific considerations for vulnerable groups, and the potential role of the Mediterranean diet as a regional model of healthy and sustainable eating during increasingly hot summers. The review also aims to identify the nature and directness of the available evidence, temper recommendations that are not supported by direct heatwave-specific studies, and highlight priorities for future research and heat-health action planning.

## 2. Materials and Methods

This narrative review synthesized current evidence on the role of diet and hydration in preventing heat-related health risks in the context of climate change, with particular attention to thermoregulation, hydration status, electrolyte balance, dietary patterns, vulnerable populations, Mediterranean diet, sustainability, and public health implications. The review was designed as a structured narrative review and was not intended to provide an exhaustive systematic review, meta-analysis, or PRISMA-ScR evidence map. A narrative approach was selected because the topic is broad, interdisciplinary, and supported by heterogeneous evidence from climate-health research, physiology, nutrition, occupational and sports medicine, geriatrics, pediatrics, public health, and sustainability science. The review aimed to provide an integrative overview of the literature, identify knowledge gaps, and outline future research and public health priorities rather than perform a quantitative synthesis.

A structured literature search was conducted using PubMed/MEDLINE, Scopus, Web of Science, Google Scholar, and institutional websites of relevant international and national organizations. The final literature search and update were completed on 30 June 2026. Peer-reviewed articles, reviews, systematic reviews, meta-analyses, position statements, consensus documents, reports, fact sheets, and public health guidance documents were considered. The search was limited to publications in English and Italian. Publications from 2010 onward were prioritized, while older landmark studies were retained when considered essential for defining physiological mechanisms, hydration requirements, heat-related health risks, or public health guidance.

Search strategies were adapted to each database and are reported in Supplementary Table S1. Search terms combined concepts related to climate change, extreme heat, heatwaves, thermoregulation, hydration, dehydration, electrolyte balance, diet, nutrition, Mediterranean diet, sustainability, food safety, vulnerable populations, and heat-health action plans. The main search domains were: (i) climate change, heatwaves, and health; (ii) heat exposure, thermoregulation, and physiological responses; (iii) hydration, dehydration, and electrolyte balance; (iv) dietary intake, meal composition, and water-rich foods; (v) vulnerable populations; (vi) Mediterranean diet, seasonality, and sustainability; and (vii) public health guidance and heat-health action plans.

The search was complemented by citation searching of key reviews, institutional reports, and guidance documents. Reference lists of highly relevant articles were screened to identify additional sources not retrieved through the initial database searches. Institutional websites were searched to identify current guidance from international and national public health or scientific organizations relevant to climate change, heat-health prevention, hydration, food safety, and sustainable diets.

Eligible documents addressed heat-related health effects, physiological responses to heat, hydration and electrolyte balance, dietary intake during hot weather, vulnerable groups, Mediterranean diet, sustainability, food safety, or public health strategies for heatwave preparedness. Publications were excluded when they were not relevant to human health, did not meaningfully address diet, hydration, heat exposure, climate change, or sustainability, or focused exclusively on animal, agricultural, or technological topics without clear relevance to human heat-health prevention. Sources dealing only with isolated bioactive compounds, specific food by-products, or agri-food valorization were excluded unless they contributed directly to the heat-health nutrition framework.

Sources were screened first by title and abstract, and then by full text when relevance was uncertain or when methodological details were needed. Because the review was narrative and not systematic, screening was not performed by duplicate independent reviewers, and no formal risk-of-bias tool was applied. The search was exploratory, iterative, and purposive, and was not intended to be exhaustive. Therefore, complete database-level counts of all records identified and excluded were not prospectively recorded. To avoid artificial precision, no retrospective PRISMA-style flow diagram was generated. The available count refers to the final body of literature retained for synthesis, which included 52 sources.

Sources were prioritized according to thematic relevance, scientific quality, directness of evidence, population relevance, recency, and contribution to the main objectives of the review. Thematic relevance was assessed according to whether each source contributed to one or more of the main domains of the review: heat exposure, thermoregulation, hydration, electrolyte balance, dietary intake, meal composition, vulnerable populations, Mediterranean dietary patterns, food safety, sustainability, or heat-health adaptation. Scientific quality was considered descriptively according to study design, methodological transparency, source reliability, population relevance, and consistency with existing physiological, clinical, nutritional, or public health evidence.

The nature and directness of the evidence were graded narratively rather than formally. Evidence was categorized as: (i) direct human heat-exposure or heatwave evidence; (ii) evidence extrapolated from sports or occupational heat settings; (iii) general nutrition evidence; (iv) mechanistic or physiological plausibility; and (v) public-health guidance or expert consensus. Greater interpretive weight was assigned to direct human evidence on heat exposure, hydration status, electrolyte balance, and heat-related outcomes. Evidence from sports nutrition, occupational heat exposure, controlled physiological studies, Mediterranean diet research, or general nutrition literature was used mainly to support mechanistic interpretation or cautious recommendations and was not treated as direct proof of heatwave-specific clinical efficacy.

When direct evidence on diet during heatwaves was limited, physiologically plausible evidence from related fields was integrated and interpreted cautiously. The selected literature was synthesized into thematic sections covering climate change and heatwaves, physiological responses to heat, hydration and electrolytes, dietary intake during high-temperature periods, vulnerable groups, Mediterranean diet and sustainability, public health implications, limitations, future perspectives, and conclusions. Findings were interpreted to identify practical implications for dietary guidance, hydration strategies, vulnerable populations, and heat-health action plans.

Use of generative artificial intelligence. During the preparation of this manuscript, ChatGPT, GPT-5.5 Thinking version (OpenAI, San Francisco, CA, USA), was used only to assist with figure development and graphical organization. The tool was not used to select literature, extract data, assess evidence, or generate unsupported scientific conclusions. All outputs were reviewed, verified, and edited by the author, who takes full responsibility for the content of the manuscript.

## 3. Evidence Framework for Nutrition and Hydration During Heat Exposure

To avoid overstatement of the available evidence, the recommendations discussed in this review were interpreted according to the nature and directness of the supporting literature. Five evidence categories were considered: direct human heat-exposure or heatwave evidence; evidence extrapolated from sports or occupational heat settings; general nutrition evidence; mechanistic or physiological plausibility; and public-health guidance or expert consensus.

Direct human heat-exposure evidence was given the greatest interpretive weight when available, particularly for hydration status, dehydration risk, heat-related illness, occupational heat strain, and vulnerable populations. Evidence from sports nutrition and occupational heat research was considered highly informative for sweat losses, fluid replacement, sodium balance, acclimatization, and exertional heat stress, but it was not automatically generalized to sedentary individuals, older adults, patients with chronic disease, or socially vulnerable populations exposed to domestic heatwaves.

General nutrition evidence was used to support broader recommendations on diet quality, water-rich foods, alcohol, sugar-sweetened beverages, Mediterranean dietary patterns, and sustainability. However, these data were interpreted cautiously when they did not directly assess heat exposure, thermoregulation, hydration status, or heat-related clinical outcomes. Similarly, mechanistic evidence on oxidative stress, inflammation, endothelial function, gastrointestinal tolerance, gut microbiota, or bioactive compounds was considered useful for biological interpretation, but not sufficient to support heatwave-specific dietary claims.

This framework was applied throughout the review to distinguish recommendations with stronger direct or consensus support from those based mainly on extrapolation or physiological plausibility. Accordingly, hydration and electrolyte guidance during heat exposure is presented as having stronger physiological and public-health support, whereas recommendations on meal size, macronutrient composition, Mediterranean diet adherence, polyphenols, and broader physiological resilience are presented as plausible but not yet directly demonstrated in real-world heatwave intervention studies.

The main recommendations discussed in this review and the nature and directness of their supporting evidence are summarized in Table 1.

## 4. Climate Change, Heatwaves, and Heat-Health Context

Climate change has substantially increased the probability, intensity, and duration of extreme heat events, making heatwaves one of the most visible and health-relevant manifestations of global warming [30]. In this review, heatwaves are considered as periods of abnormally high temperatures that last several days, although definitions vary according to geographic region, temperature thresholds, humidity, duration, and local acclimatization patterns [1,5]. Heat-related risk depends not only on outdoor temperature, but also on age, health status, housing quality, indoor exposure, access to cooling, occupational conditions, social vulnerability, and behavioral adaptation [1,5,24,25].

The public health relevance of heatwaves is supported by epidemiological evidence linking high ambient temperatures to excess morbidity and mortality. Heat exposure has been associated with cardiovascular events, renal dysfunction, respiratory exacerbations, heat exhaustion, heat stroke, adverse pregnancy outcomes, occupational injuries, reduced work capacity, and mental health effects [1,2,31]. These outcomes are mediated by several physiological pathways, including cardiovascular strain, sweating, dehydration, electrolyte imbalance, impaired renal perfusion, systemic inflammation, and reduced thermoregulatory efficiency [2,14,31]. Therefore, heat-related health effects are not limited to classical heat illness, but also include the worsening of pre-existing chronic conditions and increased pressure on health and social-care systems [2,24,25].

Europe and the Mediterranean area are particularly relevant contexts for heat-health prevention. Severe European heatwaves, including the 2003 event and the summer of 2022, have demonstrated the capacity of extreme heat to generate substantial excess mortality, especially among older adults and populations living in southern European countries [32,33]. These observations are relevant for Mediterranean regions, where high baseline summer temperatures, population aging, urbanization, and recurrent heatwaves may increase the need for targeted prevention strategies [30,33]. However, the Mediterranean and European focus of this review should be interpreted as a regional perspective rather than as a universal model applicable to all climatic and cultural settings.

The burden of heat is not distributed equally across the population. Older adults, children, pregnant women, outdoor workers, athletes, socially isolated individuals, people experiencing homelessness, and individuals with cardiovascular, renal, respiratory, metabolic, or psychiatric disorders are at increased risk [1,2,5]. Socioeconomic vulnerability further modifies heat-related risk through poor housing conditions, limited access to air conditioning or safe water, occupational exposure, reduced access to green spaces, food insecurity, and barriers to health care and public health messaging [5,24,25]. These factors are directly relevant to nutrition and hydration because they influence fluid intake, food access, food storage, appetite, medication safety, and the ability to implement heat-health recommendations.

From a public health perspective, heatwaves require integrated prevention strategies that combine early-warning systems, urban planning, housing interventions, occupational protection, social care, health communication, and targeted support for high-risk groups [4,5]. The World Health Organization Regional Office for Europe emphasizes the role of heat-health action plans as a key adaptation measure, including meteorological warning systems, coordinated emergency responses, communication campaigns, and interventions directed at vulnerable groups [5]. However, most heat-health plans still address nutrition and hydration mainly through general messages, such as drinking water and avoiding alcohol, rather than through structured, population-specific nutritional guidance.

This represents an important gap for nutritional science and public health practice. Heat exposure directly affects fluid balance, sweating, electrolyte losses, appetite, food choices, food safety, and the feasibility of maintaining adequate nutrition during hot weather [6,7,8,14]. Within this framework, hydration and dietary guidance should be considered as supportive components of heat-health prevention, while recognizing that the strength of evidence varies across recommendations. Hydration guidance is supported by stronger physiological and public-health evidence, whereas recommendations on meal composition, dietary patterns, and Mediterranean diet adherence are often based on indirect evidence, extrapolation, or biological plausibility rather than direct heatwave-specific intervention studies.

Overall, climate change is transforming heat from a seasonal discomfort into a recurring public health threat. Within this context, nutrition and hydration may contribute to heat-health preparedness when integrated with cooling strategies, clinical care, occupational protection, social support, and public health action plans. However, they should be presented as one component of a broader adaptation strategy, not as stand-alone solutions for preventing heat-related morbidity and mortality.

## 5. Physiological Responses to High Ambient Temperatures

The maintenance of core body temperature within a narrow physiological range is essential for human survival. Under conditions of high ambient temperature, the body activates thermoregulatory responses aimed at dissipating excess heat and preserving internal homeostasis. Heat exchange occurs through radiation, conduction, convection, and evaporation; however, when ambient temperature approaches or exceeds skin temperature, evaporative heat loss through sweating becomes the dominant cooling mechanism [3,31]. This process depends on sweat production, skin blood flow, hydration status, air movement, clothing, humidity, and the individual capacity for heat acclimatization [3,14]. In this review, heat acclimatization refers to adaptive responses that develop after repeated exposure to natural hot environments, whereas heat acclimation refers to similar adaptations induced under controlled or artificial heat exposure conditions.

Cutaneous vasodilation and sweating are the main physiological responses to heat exposure. Vasodilation increases skin blood flow and facilitates heat transfer from the body core to the periphery, but it also increases cardiovascular demand [3,31]. Sweating enables evaporative cooling, but it causes progressive loss of water and electrolytes, mainly sodium and chloride [3,14]. If these losses are not adequately replaced, hypohydration may develop, reducing plasma volume, increasing cardiovascular strain, impairing renal perfusion, and limiting thermoregulatory efficiency [2,3,14]. Here, hypohydration refers to a body water deficit, whereas dehydration refers to the dynamic process of losing body water; although these terms are often used interchangeably in public health guidance, this distinction is useful when interpreting physiological evidence.

**Figure 1:**
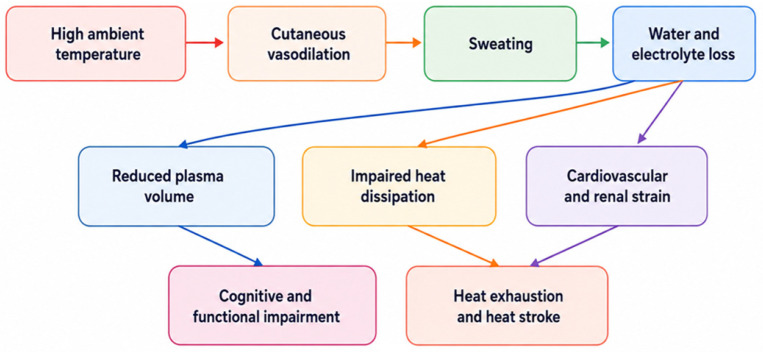
Physiological pathways linking extreme heat to health risk. High ambient temperature activates cutaneous vasodilation and sweating as thermoregulatory responses. When sweating leads to water and electrolyte loss, reduced plasma volume, impaired heat dissipation, cardiovascular and renal strain, cognitive and functional impairment, heat exhaustion, and heat stroke may occur, particularly in vulnerable individuals or during prolonged exposure.

These physiological pathways are summarized in Figure 1.

The consequences of heat exposure extend beyond fluid balance. High temperatures may affect renal function, cognitive performance, functional capacity, and cardiovascular stability, particularly when dehydration, prolonged exposure, or physical exertion are present [2,3,31]. Repeated or prolonged heat stress, especially during heavy work in hot environments, has been associated with kidney stress and may contribute to acute kidney injury or chronic kidney disease risk in highly exposed occupational groups [2,24]. This evidence is particularly relevant for outdoor workers and other highly exposed populations, but it should not be automatically generalized to all individuals exposed to domestic heatwaves.

High ambient temperatures may also influence appetite, meal tolerance, and gastrointestinal comfort. Heat exposure is commonly associated with reduced appetite and a preference for lighter meals, although individual responses vary according to age, activity level, hydration status, health status, and cultural dietary habits. The suggestion to consume smaller and more digestible meals during hot weather is based mainly on physiological plausibility and public health guidance, rather than on direct trials demonstrating improved heat-related clinical outcomes. Large or energy-dense meals may increase diet-induced thermogenesis and digestive burden, but the direct relevance of meal size and macronutrient composition to heat tolerance remains insufficiently studied [18,19].

Electrolyte balance is relevant during heat exposure, but its importance depends on context. For most healthy individuals exposed to usual summer heat, regular water intake and a balanced diet are generally sufficient to maintain hydration and electrolyte status [6,7,8]. In contrast, individuals experiencing prolonged or intense sweating, such as outdoor workers, endurance athletes, or people exposed to sustained heat without adequate cooling, may require more specific attention to sodium replacement and fluid timing [3,14]. Excessive water intake without adequate sodium replacement during prolonged exertion may increase the risk of exercise-associated or dilutional hyponatremia, whereas insufficient fluid intake increases the risk of dehydration and heat-related illness [14]. Therefore, hydration advice should be adapted to exposure duration, physical activity, sweat losses, clinical status, medication use, and the risk of both underhydration and overhydration.

The progression from physiological heat strain to heat-related illness occurs when thermoregulatory mechanisms are overwhelmed. Mild manifestations may include thirst, fatigue, weakness, dizziness, headache, muscle cramps, and reduced exercise tolerance. More severe manifestations include heat exhaustion and heat stroke, a life-threatening condition characterized by severe hyperthermia and central nervous system dysfunction [2,31]. In suspected heat stroke, hydration and nutritional measures should be considered preventive or supportive only and must not delay emergency medical care, rapid cooling, and right clinical management.

Overall, the physiological response to high ambient temperatures depends on the interaction between environmental conditions, hydration status, cardiovascular capacity, renal function, behavioral adaptation, physical activity, clinical vulnerability, and nutritional intake. These mechanisms provide a biological rationale for integrating hydration and dietary advice into heat-health prevention, but they should be distinguished from direct clinical evidence, which remains limited for many specific dietary recommendations during real-world heatwaves.

## 6. Hydration, Electrolytes, and Prevention of Dehydration

Hydration is one of the most immediate and modifiable components of heat-health prevention during exposure to high ambient temperatures. In hot environments, the maintenance of water and electrolyte balance is essential to support sweating, skin blood flow, cardiovascular stability, renal function, cognitive performance, and thermoregulation [3,14]. This recommendation is supported by strong physiological rationale, public health guidance, and evidence from controlled heat exposure, occupational, and sports settings, although population-based intervention studies during real-world heatwaves remain limited. When fluid intake is insufficient to compensate for water losses through sweat, respiration, urine, and feces, a progressive reduction in total body water may occur, increasing physiological strain and the risk of heat-related illness [2,3,14,31].

Total water requirements vary according to age, sex, body size, diet, physical activity, environmental temperature, humidity, clothing, health status, and medication use. The European Food Safety Authority established adequate intakes for total water, including water from beverages and foods, of approximately 2.0 L/day for adult women and 2.5 L/day for adult men under conditions of moderate environmental temperature and moderate physical activity [6]. Similarly, the Institute of Medicine established dietary reference intakes for water and electrolytes, emphasizing that water requirements are influenced by environmental exposure, physical activity, and individual variability [10]. These reference values provide useful baseline guidance, but they should not be interpreted as fixed heatwave-specific targets, because fluid needs may increase substantially during hot weather, fever, physical exertion, or prolonged sweating.

During hot weather, dehydration can develop through inadequate fluid intake, excessive water loss, or both. Low-intake dehydration may be particularly relevant among older adults, individuals with impaired thirst, people with cognitive or functional limitations, and those dependent on caregivers [11,12]. Sweat-loss dehydration is more common in outdoor workers, athletes, and physically active individuals exposed to high heat load for prolonged periods [3,14]. These mechanisms may overlap during heatwaves, when reduced appetite, limited mobility, social isolation, gastrointestinal symptoms, fever, restricted access to safe water, or increased sweating may contribute to clinically relevant dehydration.

The physiological effects of dehydration are closely linked to reductions in plasma volume and increases in plasma osmolality. Even modest fluid deficits can increase cardiovascular strain by reducing venous return, increasing heart rate, and limiting the ability to sustain both skin blood flow and perfusion of vital organs [3,14]. Dehydration also reduces sweating efficiency and may accelerate the rise in core body temperature during heat exposure or physical exertion [3,14]. In vulnerable individuals, these changes may contribute to dizziness, orthostatic hypotension, falls, acute kidney stress, cognitive impairment, and exacerbation of chronic disease [2,5,11]. Hydration should therefore be interpreted as a preventive and supportive strategy; suspected heat stroke requires urgent medical evaluation, rapid cooling, and emergency management.

Electrolyte balance is another essential component of hydration during heat exposure. Sweat contains water and electrolytes, primarily sodium and chloride, with smaller amounts of potassium, magnesium, and calcium [3,14]. Sodium helps maintain extracellular fluid volume, supports thirst mechanisms, and contributes to fluid retention after ingestion [10,14]. For most healthy individuals exposed to usual summer heat, a balanced diet and regular fluid intake are generally sufficient to maintain electrolyte balance [6,7,8]. In contrast, prolonged sweating, heavy physical work, endurance exercise, or repeated heat exposure may increase sodium losses and require individualized replacement strategies [3,14]. This evidence is stronger in sports and occupational heat settings than in sedentary populations exposed to domestic heatwaves.

Hydration recommendations should distinguish between the general population, individuals with high sweat losses, and people with clinical vulnerabilities. For the general population, public health authorities recommend drinking water regularly, increasing fluid intake during hot weather, avoiding alcohol, and moderating highly sugary or caffeinated beverages [1,7,8]. These recommendations are particularly important because thirst may not be a sufficient indicator of hydration needs in older adults or during sustained heat exposure [11,12]. Practical strategies include keeping water visible and accessible, drinking small amounts frequently, consuming water-rich foods, and monitoring simple warning signs such as dry mouth, fatigue, dizziness, confusion, or reduced urination [7,8,11]. Urine color may be used only as an approximate screening tool and may be unreliable in individuals with kidney disease, urinary disorders, use of diuretics or vitamin supplements, or altered urine concentration.

Older adults and patients with chronic diseases require particular caution. Aging is associated with reduced total body water, blunted thirst perception, impaired renal concentrating capacity, lower muscle mass, chronic disease, and polypharmacy [5,11,12]. A systematic review of interventions to improve hydration in acutely unwell or institutionalized older adults suggests that multifaceted strategies, including drink availability, preferred beverages, verbal prompts, adapted drinking vessels, and caregiver involvement, may improve fluid intake, although evidence remains heterogeneous [13]. In frail, institutionalized, or cognitively impaired individuals, scheduled drinking and caregiver-supported hydration are generally more appropriate than relying exclusively on thirst. Conversely, in people with chronic kidney disease, heart failure, advanced cardiovascular disease, dysphagia, or prescribed fluid restriction, generic advice such as “drink as much as possible” may be unsafe and should be replaced by individualized clinical guidance [2,5,10].

In physically active individuals and outdoor workers, hydration strategies should account for sweat rate, duration of exposure, work or exercise intensity, acclimatization status, clothing, and environmental conditions. Fluid replacement should aim to prevent excessive body water deficit while avoiding overhydration [14]. During prolonged activity in the heat, beverages containing sodium may improve fluid retention and help replace sweat sodium losses, particularly when sweat losses are high or exposure lasts several hours [14]. However, generalized electrolyte supplementation is not necessary for all individuals and may be inappropriate in people with hypertension, kidney disease, heart failure, or medications affecting fluid and electrolyte balance. Therefore, electrolyte replacement should be individualized rather than presented as a universal heatwave recommendation.

Excessive fluid intake also deserves attention. Exercise-associated or dilutional hyponatremia can occur when fluid intake exceeds renal excretory capacity and plasma sodium concentration becomes diluted, especially during prolonged endurance activity or sustained heat exposure with high fluid intake and inadequate sodium replacement [14]. Although this condition is less relevant for the general sedentary population during heatwaves, it is important for athletes, recreational exercisers, military personnel, and workers exposed to long periods of heat. Balanced advice should therefore emphasize drinking regularly and appropriately, replacing losses according to exposure and activity, and avoiding both underhydration and overhydration.

The type of beverage may also influence short-term fluid retention. Water remains the most appropriate first-line beverage for the general population during hot weather because it is widely available, energy-free, and effective for replacing usual fluid losses [7,8]. Experimental work on the beverage hydration index has shown that drinks differ in their short-term fluid retention capacity according to electrolyte content, energy density, and macronutrient composition [17]. This evidence may be relevant in selected contexts, such as institutional care, prolonged occupational exposure, or post-exercise rehydration, but it should not be used to promote sugar-sweetened or alcoholic beverages as hydration strategies during heatwaves.

Food also contributes to hydration. A substantial proportion of total water intake can derive from foods, particularly fruits, vegetables, soups, milk, yogurt, and other high-moisture foods [6,10]. During periods of high temperature, water-rich foods may contribute to fluid intake while also providing potassium, vitamins, fiber, and other nutrients. However, food-derived water should complement rather than replace access to safe drinking water, especially during heatwaves, physical exertion, or prolonged outdoor exposure. The role of water-rich foods is therefore supportive for hydration and diet quality, but direct evidence that they reduce heat-related clinical outcomes remains limited.

**Figure 2:**
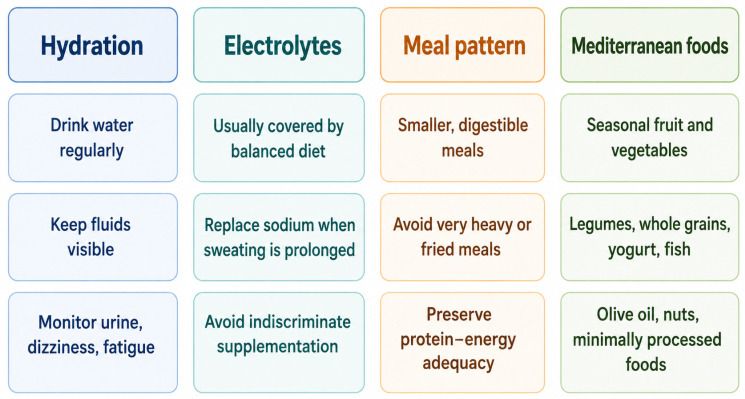
Diet and hydration strategies during high-temperature periods. Key recommendations include regular water intake, context-specific electrolyte replacement, lighter meals, and Mediterranean foods rich in water and nutrients.

Key dietary and hydration strategies during high-temperature periods are summarized in Figure 2.

Overall, hydration and electrolyte balance represent a core interface between nutrition, physiology, and heat-health prevention. The available evidence supports regular water intake, attention to vulnerable groups, avoidance of alcohol, use of water-rich foods as supportive dietary components, and individualized electrolyte replacement in conditions of prolonged or intense sweating [6,7,8,10,11,12,13,14,17]. However, the strength and directness of evidence vary across recommendations: water intake and fluid replacement are supported by stronger physiological and public-health evidence, whereas broader dietary recommendations during heatwaves often rely on indirect evidence, expert guidance, or biological plausibility. Hydration should therefore be framed as a structured, context-specific preventive strategy rather than as a generic instruction to increase fluid intake for all individuals.

## 7. Dietary Intake During High-Temperature Periods: Meal Composition and Diet Quality

Dietary intake during periods of high ambient temperature should be considered not only in terms of energy and nutrient adequacy, but also in relation to hydration, gastrointestinal tolerance, food safety, and overall diet quality. Although general nutritional principles support the recommendation of lighter, water-rich, nutrient-dense meals during hot weather, direct evidence linking specific meal patterns to improved heat-related clinical outcomes remains limited [1,6,7,8]. Therefore, dietary recommendations in this area should be interpreted mainly as supportive and physiologically plausible strategies rather than as fully established heatwave-specific protocols.

High temperatures may influence appetite, food preferences, and eating patterns. Many individuals report reduced appetite and a preference for smaller meals, cold dishes, fruit, vegetables, and beverages during hot weather. This response may reduce digestive discomfort, but it can also become problematic in older adults, frail individuals, patients with chronic diseases, and people with limited access to food. In these groups, reduced intake may contribute to weakness, dehydration risk, sarcopenia, impaired immune function, and undernutrition [5,11,12,13]. Thus, advice to “eat light” should not be interpreted as dietary restriction, particularly in vulnerable populations.

Meal size and macronutrient composition can influence postprandial thermogenesis. Diet-induced thermogenesis is affected by total energy intake, macronutrient composition, meal timing, and eating pattern [18,19]. Larger meals may increase digestive workload and thermal discomfort, providing a plausible rationale for smaller and more digestible meals during the hottest hours of the day. However, evidence supporting specific recommendations on meal size, protein intake, carbohydrate distribution, or fat intake during real-world heatwaves remains indirect. In particular, protein intake should not be reduced indiscriminately, because adequate protein is essential for muscle maintenance, immune function, recovery from physical work, and healthy aging. For older adults and individuals at risk of malnutrition, the priority should be to distribute protein across smaller, tolerable meals rather than to restrict it.

Carbohydrate and electrolyte-containing beverages may have a role during prolonged endurance exercise or heavy occupational heat exposure, but their use should not be generalized to the sedentary population [14,20]. Frequent consumption of sugar-sweetened beverages should not be promoted as a hydration strategy, especially in individuals with obesity, diabetes, or cardiometabolic risk [7,8]. Similarly, sodium replacement may be appropriate in conditions of prolonged or intense sweating, but this does not justify the generalized promotion of salty or ultra-processed foods during hot weather [14]. For most healthy individuals, a balanced diet combined with adequate water intake is sufficient to maintain electrolyte balance during usual summer conditions [6,7,8].

Water-rich foods represent a useful dietary component during high-temperature periods. Fruits, vegetables, soups, milk, yogurt, and other high-moisture foods can contribute to total water intake while also providing potassium, vitamins, fiber, and other nutrients [6,10,17]. In Mediterranean settings, seasonal fruits and vegetables such as tomatoes, cucumbers, leafy greens, zucchini, peaches, citrus fruits, melon, and watermelon may support both hydration and diet quality. However, food-derived water should complement, not replace, regular access to safe drinking water, especially during heatwaves, physical exertion, or prolonged outdoor exposure. The evidence for water-rich foods is stronger as a contribution to total fluid intake and diet quality than as direct proof of reduced heat-related morbidity.

Micronutrients and bioactive compounds may contribute to general metabolic health, but their specific role in heat adaptation remains uncertain. Heat stress has been associated with oxidative stress, inflammation, gastrointestinal barrier alterations, immune changes, and metabolic disturbances, leading to interest in antioxidant vitamins, polyphenols, trace elements, amino acids, and electrolytes [20]. At present, however, evidence does not support broad supplementation recommendations for the general population during heatwaves. Instead, dietary advice should prioritize whole foods, including fruits, vegetables, legumes, whole grains, nuts, seeds, fermented dairy products when appropriate, and other minimally processed foods. Supplementation should be reserved for documented deficiencies, specific clinical conditions, heavy sweating, or high-performance sport, preferably under professional guidance.

The Mediterranean diet may provide a useful regional model for dietary advice during increasingly hot summers because it emphasizes seasonal plant foods, water-rich fruits and vegetables, legumes, whole grains, olive oil, culinary simplicity, and minimally processed foods [9,21,22]. Nevertheless, Mediterranean diet adherence has not been directly demonstrated to improve heat tolerance, hydration status, or heat-related clinical outcomes. Therefore, it should be interpreted as a culturally rooted example of a healthy and potentially sustainable dietary pattern, rather than as a proven heat-protective intervention. Similar principles may be adapted to other cultural and climatic contexts using locally available, affordable, water-rich, and minimally processed foods.

Food safety is an essential component of nutrition during high-temperature periods. Elevated temperatures can accelerate microbial growth and increase the risk of foodborne disease when foods are improperly stored, transported, prepared, or consumed [23]. This issue is particularly relevant during heatwaves, power outages, outdoor meals, institutional food service, and situations in which refrigeration is limited. Public health advice should therefore include basic food-safety measures, such as keeping clean, separating raw and cooked foods, cooking thoroughly, keeping food at safe temperatures, and using safe water and raw materials [23]. Food safety is especially important for children, older adults, pregnant women, and immunocompromised individuals.

Dietary recommendations during high-temperature periods should therefore be differentiated by population group. For healthy adults, the main priorities are adequate water intake, water-rich foods, moderate meal size, avoidance of alcohol, limitation of sugar-sweetened beverages, and maintenance of overall diet quality [7,8]. For older adults, frail individuals, and patients with chronic diseases, preventing both dehydration and undernutrition is essential [11,12,13]. For outdoor workers and athletes, meal timing, carbohydrate availability, sodium replacement, and fluid planning may be required according to workload and sweat losses [14,20]. For children and pregnant women, guidance should emphasize safe hydration, nutrient-dense foods, food safety, and caregiver-supported monitoring during heatwaves [1,5,23].

Overall, dietary strategies during high-temperature periods should not be reduced to generic advice to “eat light”. A more appropriate approach is to promote nutritionally adequate, water-rich, digestible, and minimally processed meals that support hydration and diet quality without compromising energy, protein, or micronutrient intake. However, recommendations on meal composition, meal timing, macronutrient distribution, Mediterranean diet adherence, and bioactive compounds should be presented cautiously, because they are supported mainly by physiological plausibility, general nutrition evidence, or extrapolation from related fields rather than by direct heatwave-specific intervention studies.

## 8. Population-Specific Considerations for Vulnerable Groups

Heat-related nutritional and hydration guidance should be adapted to population-specific physiological, clinical, occupational, and social conditions. Older adults, children, pregnant women, individuals with chronic diseases, outdoor workers, athletes, socially isolated individuals, and socioeconomically disadvantaged groups may have different risks, needs, and capacities to implement general heat-health recommendations [1,2,5,11,12,13,15,16,26,27,28,29].

Older adults are particularly vulnerable because of reduced total body water, impaired thirst perception, lower renal concentrating capacity, chronic disease, polypharmacy, reduced mobility, and risk of undernutrition [11,12,13,26]. In this group, scheduled drinking, caregiver-supported hydration, water-rich foods, and smaller nutrient-dense meals may be useful. However, advice to increase fluid intake should be individualized in the presence of heart failure, chronic kidney disease, dysphagia, or prescribed fluid restriction [2,5,10].

Children and pregnant women also require specific attention. Children depend on caregivers for access to fluids, food, shade, and recognition of early heat-related symptoms [1,27,28]. Pregnant women may be more vulnerable because of increased cardiovascular and metabolic demands, and heat exposure has been associated with adverse maternal, fetal, and neonatal outcomes [29]. In these groups, guidance should emphasize safe hydration, nutrient-dense foods, food safety, and avoidance of alcohol.

Outdoor workers and athletes may experience high sweat losses because of physical activity, clothing, environmental exposure, and prolonged heat load [3,14,15,16]. In these settings, fluid timing, recovery hydration, and sodium replacement may be relevant, especially during prolonged or intense sweating. However, recommendations derived from sports and occupational heat research should not be generalized automatically to sedentary individuals or medically vulnerable populations.

Socioeconomically disadvantaged populations may be unable to follow standard recommendations because of limited access to safe drinking water, refrigeration, fresh foods, cooling environments, health care, or reliable information [1,5,24,25]. Therefore, nutritional advice for heatwaves should be accompanied by structural support, including public water access, food assistance, cooling centers, community outreach, and targeted monitoring of isolated or high-risk individuals.

## 9. Mediterranean Diet, Seasonality, and Environmental Sustainability

The Mediterranean diet may provide a useful regional model for dietary advice during increasingly hot summers because it emphasizes seasonal plant foods, water-rich fruits and vegetables, legumes, whole grains, olive oil, culinary simplicity, and minimally processed foods [9,21,22].

**Figure 3:**
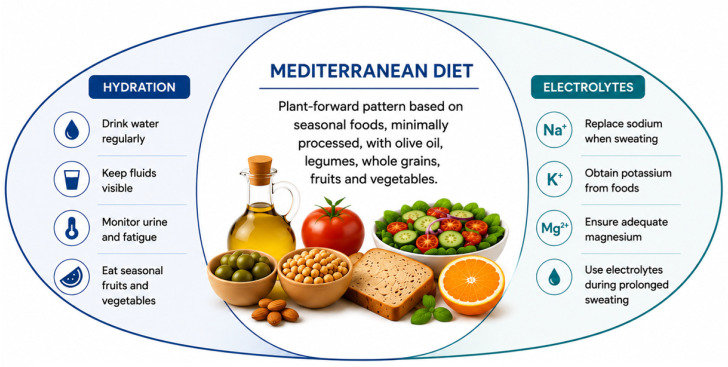
Mediterranean diet, seasonality, and environmental sustainability as a regional framework. The figure summarizes the Mediterranean diet as a plant-forward dietary pattern based on seasonal, minimally processed foods, olive oil, legumes, whole grains, fruits, and vegetables. It highlights its links with dietary quality, water-rich foods, cultural acceptability, local food systems, and environmental sustainability. These dimensions should be interpreted as part of a regional food-system perspective and not as direct evidence of heat-specific protection.

This regional framework is summarized in Figure 3.

However, its role in heat adaptation should be interpreted cautiously, because direct evidence linking Mediterranean diet adherence to improved heat tolerance, hydration status, or heat-related clinical outcomes remains limited. Therefore, the Mediterranean diet should be considered a culturally rooted example of a healthy and potentially sustainable dietary pattern, rather than a proven heat-protective intervention.

Seasonality is particularly relevant in the context of heat-health nutrition. During summer, traditional Mediterranean meals often include fresh vegetables, legumes, salads, fruit, yogurt, olive oil, herbs, cold pasta or grain-based dishes, and fish. These foods and meals may be more acceptable during hot weather than large, heavy, or highly processed meals and may help preserve diet quality when appetite is reduced [6,7,8,18,19]. Nevertheless, the recommendation to favor seasonal, water-rich, and digestible foods is supported mainly by general nutrition evidence, public health guidance, and physiological plausibility rather than by direct heatwave-specific intervention studies.

The Mediterranean diet is also relevant to environmental sustainability because it is generally more plant-forward and less dependent on red and processed meat than many Western dietary patterns [9,22,34,35,36]. This may be compatible with broader recommendations for sustainable healthy diets, which emphasize nutritional adequacy, reduced environmental impact, cultural acceptability, biodiversity protection, and accessibility [22,36]. However, the sustainability of the Mediterranean diet should not be considered automatic. Environmental impact depends on food choices, production systems, seasonality, transport, processing, packaging, refrigeration, cooking practices, affordability, and food waste [9,34,35,36].

In Mediterranean regions, climate change may also threaten the availability, price, safety, and quality of traditional foods through rising temperatures, drought, water scarcity, soil degradation, biodiversity loss, and extreme weather events [24,30,36]. Thus, the Mediterranean diet can be discussed as part of a regional food-system perspective, but not as a universal dietary solution for heatwaves. Its transferability to other climatic, cultural, socioeconomic, and food-system contexts should be interpreted with caution.

Food-waste reduction may represent an additional sustainability dimension during hot seasons, especially because high temperatures can accelerate food spoilage and increase the risk of foodborne disease when refrigeration and storage are inadequate [23]. However, specific discussions of agri-food by-products, isolated bioactive compounds, functional ingredients, or cultivar-specific examples are beyond the central heat-health objective of this review and were therefore not retained as a major focus.

Overall, the Mediterranean diet offers a useful regional framework for aligning dietary quality, seasonality, cultural acceptability, and environmental sustainability. Its relevance for heat-health nutrition lies mainly in its emphasis on seasonal, water-rich, minimally processed, and nutrient-dense foods, not in direct evidence of heat-specific protection. Future research should evaluate whether Mediterranean dietary patterns or other culturally appropriate plant-forward diets influence hydration status, dietary adequacy, and heat-related outcomes during real-world heatwaves.

## 10. Public Health Implications

The increasing frequency, intensity, and duration of heatwaves require a shift from emergency-oriented responses to integrated, preventive, and multisectoral public health strategies. Extreme heat should be considered not only an environmental exposure, but also a nutrition-sensitive public health issue, because it can affect hydration, appetite, food safety, food access, medication tolerance, functional capacity, and the risk of acute and chronic disease exacerbation [1,2,5,24]. Within this framework, nutrition and hydration should be integrated into heat-health action plans as supportive preventive components, while avoiding the impression that dietary measures alone can prevent heat-related morbidity and mortality.

Heat-health action plans are among the main public health tools for reducing heat-related morbidity and mortality. These plans typically include meteorological warning systems, risk communication, identification of vulnerable groups, health-care preparedness, surveillance, emergency response, urban adaptation, and evaluation procedures [5,37,38,39]. The updated WHO guidance on heat-health action plans emphasizes governance, warning systems, communication, health-system resilience, protection of populations at increased risk, reduction of heat exposure, heat-health surveillance, and monitoring, evaluation, and learning as core elements of effective heat preparedness [37]. Nutritional guidance can be incorporated across several of these elements, particularly communication, clinical preparedness, protection of high-risk groups, institutional protocols, occupational settings, and community outreach.

**Figure 4:**
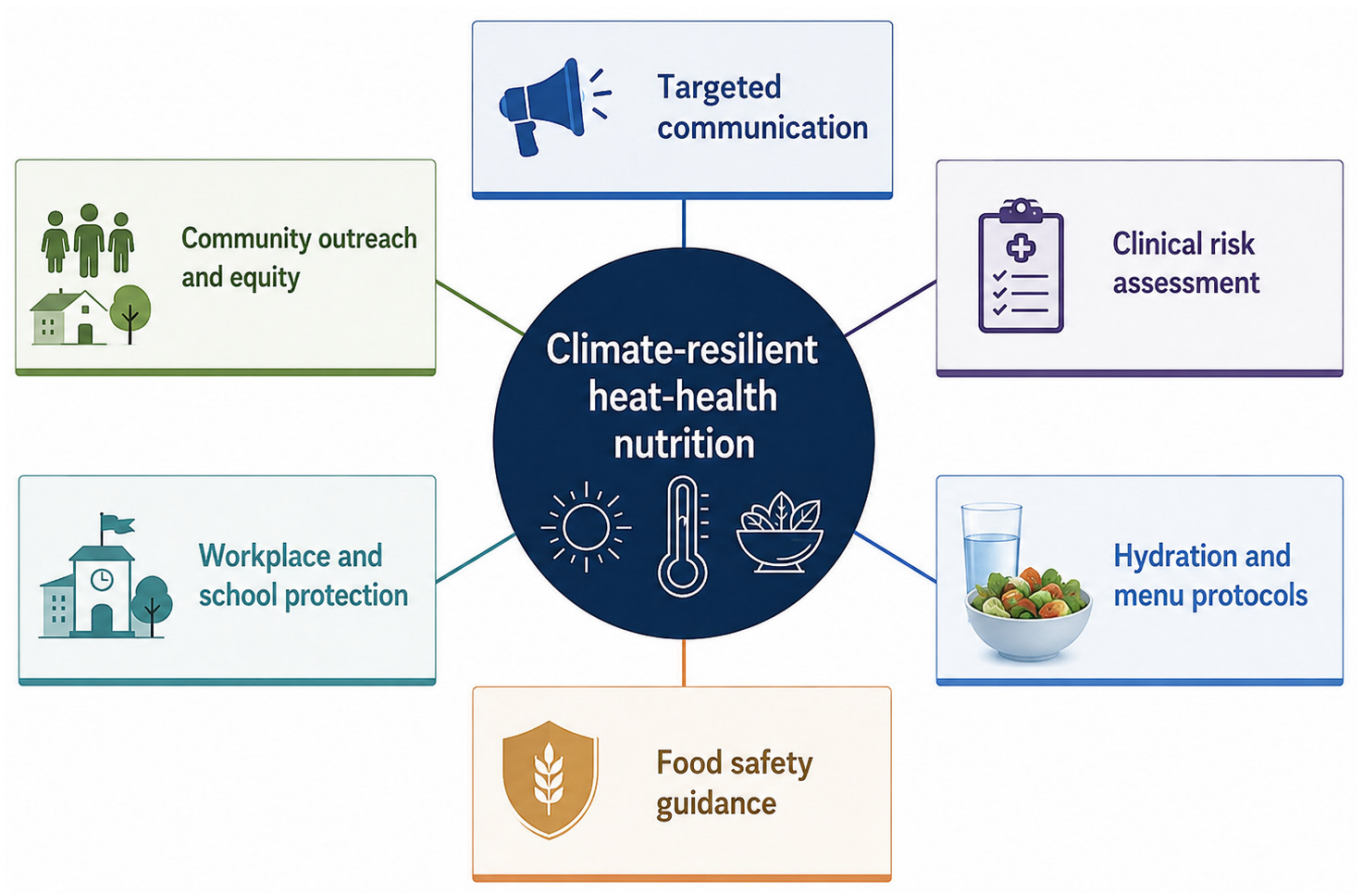
Integrating nutrition into heat-health action plans. Nutrition-sensitive heat-health action plans should combine targeted communication, clinical risk assessment, hydration and menu protocols, food safety guidance, workplace and school protection, and community outreach with an equity-oriented approach. These actions may support heat-health preparedness but should be interpreted as part of a broader adaptation strategy that also includes cooling, urban planning, occupational protection, clinical care, and emergency response.

The main domains for integrating nutrition into heat-health action plans are summarized in Figure 4.

Public health communication during heatwaves should provide clear, actionable, repeated, and population-specific messages. General recommendations may include regular water intake, avoidance of alcohol, moderation of sugar-sweetened beverages, use of water-rich foods as supportive dietary components, preference for digestible meals, and attention to food safety [7,8,23]. However, these messages should be presented with proper nuance because their evidence base differs hydration advice is supported by stronger physiological and public-health evidence, whereas recommendations on meal composition and dietary patterns are more often based on indirect evidence or physiological plausibility. Universal messages may also be insufficient or inappropriate for some groups, including older adults, patients with heart failure or chronic kidney disease, people taking heat-sensitive medications, athletes, outdoor workers, pregnant women, and children [2,5,11,12,13,14,15,16,29].

Health professionals have a central role in translating heat-health advice into individualized prevention. Primary care physicians, dietitians, nurses, pharmacists, occupational physicians, sports medicine professionals, and community health workers can identify individuals at high risk, review medication-related vulnerability, provide hydration and dietary counseling, and promote patient-level heat action plans [38,40]. Clinical encounters before and during summer periods may be used to assess risk factors such as advanced age, chronic kidney disease, heart failure, diabetes, pregnancy, cognitive impairment, social isolation, occupational exposure, and limited access to cooling or safe water [2,5,40]. In this context, nutrition counseling should be embedded in broader heat-risk assessment rather than delivered as isolated dietary advice.

Institutional and occupational settings require specific heat-nutrition protocols. Nursing homes, hospitals, rehabilitation facilities, schools, prisons, shelters, and workplaces should have predefined procedures for hydration rounds, monitoring of fluid intake, menu adaptation, access to water-rich foods, safe food storage, and recognition of dehydration or heat-related symptoms [13,23,37,40]. In long-term care facilities, protocols should not rely solely on spontaneous drinking, especially among older adults with cognitive impairment, dysphagia, mobility limitations, or reduced thirst perception [11,12,13,26]. Outdoor and heat-exposed workers require access to safe drinking water, shaded rest areas, scheduled breaks, acclimatization procedures, proper clothing, and education on early symptoms of heat strain [14,15,16,37]. For workers with prolonged or intense sweating, fluid timing and sodium replacement may be relevant, but these strategies should be individualized and should not be generalized to all populations.

Food safety should be included in heat-health planning. High temperatures can accelerate microbial growth and food spoilage, increasing the risk of foodborne disease when refrigeration, transport, preparation, or storage are inadequate [23]. Heat-health plans should therefore include food safety messages for households, schools, hospitals, nursing homes, restaurants, and community food services. These messages should address safe storage temperatures, separation of raw and cooked foods, adequate cooking, prompt refrigeration, safe drinking water, and special attention to pregnant women, children, older adults, and immunocompromised individuals [23]. Food safety is particularly important during heatwaves, power outages, outdoor meals, and institutional catering.

Equity should be a core principle of public health nutrition during heatwaves. Socioeconomically disadvantaged populations may have limited access to air conditioning, safe drinking water, refrigeration, fresh foods, health care, and reliable heat-risk information [1,5,24,25,39]. People experiencing homelessness, socially isolated older adults, migrants, low-income households, and individuals living in poorly insulated housing may be unable to implement standard recommendations. For these groups, nutritional advice must be accompanied by material support, including public water access, cooling centers, food assistance, outreach services, and community-based monitoring [37,41,42]. Without such support, heat-health communication may be ineffective and may widen existing inequalities.

Surveillance and evaluation are essential to improve heat-health interventions. Public health systems should monitor heat-related morbidity, mortality, emergency department visits, ambulance calls, occupational incidents, dehydration-related admissions, kidney injury, and foodborne disease during periods of extreme heat [5,37,38,39]. Evaluation should also assess whether communication campaigns and interventions reach vulnerable groups, improve hydration behaviors, reduce dehydration, and prevent adverse outcomes [37,43,44]. Nutrition-related indicators, such as dehydration admissions, institutional hydration monitoring, access to safe water, and foodborne illness during heatwaves, could strengthen the evaluation of heat-health action plans.

Overall, public health responses to extreme heat should move beyond individual behavioral advice and adopt a systems-based approach. Nutrition and hydration should be considered modifiable components of heat-health preparedness, but their effectiveness depends on clinical appropriateness, institutional support, social equity, environmental cooling, occupational protection, and access to safe water and adequate food. In a warming climate, integrating dietary and hydration guidance into heat-health action plans may help protect vulnerable populations, provided that these strategies are implemented as part of broader multidisciplinary adaptation policies.

## 11. Limitations of the Literature

Although the relationship between extreme heat and adverse health outcomes is supported by a large and growing body of evidence, the specific role of diet and hydration as structured components of heat-health prevention remains insufficiently investigated. Much of the available literature focuses on heat-related morbidity and mortality, thermoregulation, occupational heat strain, sports physiology, and public health preparedness [1,2,3,4,5,14,15,16,24,25,31,37,38,39,40,41,42,43,44,45,46,47,48]. In contrast, fewer studies have directly examined how dietary patterns, meal composition, water-rich foods, electrolyte intake, and nutritional status influence heat tolerance, dehydration risk, clinical outcomes, or resilience during real-world heatwaves in the general population and in vulnerable groups. The final body of literature retained for synthesis was relatively limited, reflecting the emerging and fragmented nature of research specifically addressing diet, hydration, and heatwave-related health outcomes.

The first limitation concerns the heterogeneity of heatwave definitions and exposure metrics. Heatwaves are defined differently across studies, with variation in temperature thresholds, duration, humidity, night-time temperature, geographic context, and population acclimatization [1,5,30]. Moreover, individual heat exposure is influenced not only by outdoor temperature, but also by housing quality, indoor temperature, urban heat island effects, clothing, physical activity, occupational conditions, access to cooling, and behavioral adaptation [5,24,37,38,39]. This heterogeneity limits the comparability of studies and complicates the development of standardized nutrition and hydration recommendations.

A second limitation is the limited integration of nutritional variables into heat-health research. Epidemiological studies often evaluate mortality, hospital admissions, cardiovascular events, renal outcomes, emergency department visits, or occupational injuries, but rarely include detailed dietary intake, hydration status, electrolyte balance, food access, food safety, or nutritional vulnerability as exposure or modifying variables [2,15,16,25,38,43,44]. As a result, it remains difficult to quantify the independent or interactive contribution of nutrition to heat-related outcomes.

A third limitation is the scarcity of intervention studies. Recommendations to drink water regularly, avoid alcohol, consume water-rich foods, eat smaller and more digestible meals, and adapt electrolyte replacement to sweat losses are biologically plausible and consistent with public health guidance [6,7,8,10,14]. However, few randomized or pragmatic trials have tested specific hydration and dietary strategies during real-world heatwaves. Evidence is especially limited for older adults, children, pregnant women, individuals with chronic kidney disease or heart failure, socially isolated populations, and people living in poorly cooled housing [11,12,13,26,27,28,29].

Another important limitation is the frequent extrapolation of evidence from sports and occupational settings to the general population. Studies in athletes, military personnel, and outdoor workers provide valuable information on sweat losses, fluid replacement, sodium balance, acclimatization, and heat strain [3,14,15,16]. However, these populations differ substantially from older adults, sedentary individuals, patients with chronic diseases, and socially vulnerable groups. Recommendations derived from exercise physiology should therefore be adapted carefully, especially regarding electrolyte supplementation, carbohydrate-electrolyte beverages, sodium intake, and fluid replacement targets [14].

The evidence on meal composition during high-temperature periods is also incomplete. Physiological principles suggest that large meals and meals with high thermogenic load may increase thermal discomfort, whereas smaller, water-rich, nutrient-dense meals may be more tolerable during hot weather [18,19]. However, direct evidence linking specific meal patterns, macronutrient composition, meal timing, or food temperature to heat-related outcomes remains limited. These recommendations should therefore be interpreted as supportive and physiologically plausible rather than as validated heatwave-specific protocols.

The role of the Mediterranean diet in heat adaptation also requires further investigation. The Mediterranean diet is supported by evidence for cardiometabolic health, dietary quality, cultural acceptability, and environmental sustainability [9,21,22,34,35,36,49,50]. Nevertheless, direct studies examining whether adherence to the Mediterranean diet improves heat tolerance, hydration status, or heat-related morbidity are lacking. Therefore, the Mediterranean diet should be considered a useful regional model of healthy, seasonal, plant-forward, and potentially sustainable eating, rather than a proven heat-protective intervention.

Another limitation is that this review has a predominantly Mediterranean and European public health perspective, particularly in the discussion of Mediterranean dietary patterns, sustainability, and heat-health action plans. Although evidence from broader climate-health, physiological, occupational, and public health literature was included, the transferability of some recommendations to other climatic, cultural, socioeconomic, and food-system contexts should be interpreted with caution.

Food safety is another underdeveloped area. High temperatures increase the risk of microbial growth, food spoilage, and foodborne disease, but heat-health nutrition guidance often focuses mainly on hydration rather than safe food storage, handling, and institutional food service [23]. This is relevant because heatwaves may simultaneously increase physiological vulnerability and compromise the safety and accessibility of foods, particularly in settings with limited refrigeration, power outages, or institutional catering.

Equity-related evidence gaps are also important. Many nutrition recommendations assume that individuals can access safe drinking water, fresh foods, refrigeration, cooking facilities, and clinical advice. However, heat vulnerability is strongly shaped by income, housing, occupation, social isolation, access to water, access to healthy foods, and access to health care [1,5,24,25,37,38,39]. Dietary and hydration advice may therefore be difficult to implement in precisely those populations at highest risk.

Methodologically, narrative reviews are useful for synthesizing complex and interdisciplinary topics, especially when evidence is heterogeneous and spans physiology, nutrition, public health, climate science, and sustainability [51]. However, narrative reviews are limited by potential selection bias, the absence of formal risk-of-bias assessment, and the lack of quantitative synthesis. Although this review used a structured narrative approach and explicitly considered the nature and directness of evidence, it was not designed as a systematic or scoping review.

Overall, the current literature supports the biological plausibility and public health relevance of hydration and dietary strategies during high-temperature periods, but major evidence gaps remain. More rigorous, population-specific, and context-sensitive research is needed to determine which nutritional interventions are most effective, for whom, under which environmental conditions, and through which mechanisms.

## 12. Future Perspectives

Future research should improve the assessment of heat exposure by moving beyond ambient outdoor temperature alone. Studies should incorporate indoor temperature, humidity, personal exposure monitoring, heat index, wet-bulb globe temperature, and time-activity patterns to better characterize the real heat burden experienced in homes, workplaces, institutions, and urban environments [5,37,38,39].

Nutritional variables should be more systematically integrated into heat-health research. Observational studies should include dietary assessment, fluid intake, biomarkers of hydration, renal function, electrolyte status, nutritional status, medication use, food access, and food safety as part of heat-risk models [2,15,16,25,38,43,44]. Such data would help clarify whether nutrition modifies the relationship between heat exposure and clinical outcomes.

Pragmatic intervention studies are particularly needed. Feasible strategies to test include scheduled hydration protocols, individualized electrolyte strategies for high-sweat populations, meal timing, water-rich meal plans, institutional menu adaptation, and community-based food and water support [11,12,13,14,15,16,37,40,41,42,45]. These studies should prioritize vulnerable settings such as nursing homes, hospitals, schools, workplaces, and socially disadvantaged communities.

Future studies should also distinguish more clearly among domestic heat exposure, occupational heat exposure, exercise-induced heat stress, and heat exposure in clinical or institutional settings [3,14,15,16]. These contexts differ substantially in sweat losses, fluid needs, electrolyte requirements, clinical risks, and feasibility of intervention.

The Mediterranean diet and other culturally appropriate plant-forward dietary patterns should be studied cautiously in relation to heat adaptation. Future research should evaluate whether seasonal, water-rich, minimally processed dietary patterns influence hydration status, dietary adequacy, and heat-related outcomes during real-world heatwaves [9,21,22,34,35,36]. Such studies should account for regional dietary diversity, affordability, food access, cultural preferences, and environmental sustainability.

Food safety and equity should become central components of heat-health nutrition research and policy. Future heat-health action plans should include food safety indicators, particularly in nursing homes, hospitals, schools, shelters, and households with limited refrigeration or during power outages [23,37]. Equity-focused studies should evaluate whether hydration and dietary interventions are feasible, affordable, culturally acceptable, and effective among low-income households, migrants, people experiencing homelessness, socially isolated older adults, and communities living in urban heat islands [1,5,24,25,37,38,39,40,41,42,45].

Finally, this field would benefit from more focused systematic reviews and, where possible, meta-analyses on specific questions, such as hydration interventions in older adults during heatwaves, electrolyte strategies for outdoor workers, food safety interventions during extreme heat, and nutrition-sensitive components of heat-health action plans [13,15,41,42,43,45,51].

Addressing these priorities will be essential to develop evidence-based dietary guidance for increasingly hot summers and to integrate nutrition into climate-resilient public health systems [52].

## 13. Conclusions

Climate change is increasing the frequency, intensity, and duration of extreme heat events, transforming heatwaves into a recurrent public health challenge with important implications for nutrition, hydration, and vulnerable populations. High ambient temperatures affect human health through thermoregulatory strain, sweating, dehydration, electrolyte imbalance, cardiovascular and renal stress, inflammation, and impaired cognitive and functional performance [1,2,3,4,5,14,24,25,31]. These effects are particularly relevant for older adults, children, pregnant women, people with chronic diseases, outdoor workers, athletes, socially isolated individuals, and socioeconomically disadvantaged groups [1,2,5,11,12,13,15,16,26,27,28,29].

Hydration represents the most directly supported nutritional component of heat-health prevention. Regular water intake, avoidance of alcohol, moderation of sugar-sweetened beverages, use of water-rich foods as supportive dietary components, and individualized electrolyte replacement during prolonged or intense sweating may help reduce dehydration risk and physiological heat strain [6,7,8,10,11,12,13,14,17]. However, hydration advice should be context-specific and should avoid both underhydration and overhydration, particularly in individuals with chronic kidney disease, heart failure, medication-related vulnerability, or high occupational or athletic sweat losses.

Dietary strategies during high-temperature periods should aim to preserve nutritional adequacy while reducing unnecessary digestive and metabolic burden. Smaller, digestible, nutrient-dense meals and water-rich, minimally processed foods may support diet quality and gastrointestinal tolerance during hot weather [6,7,8,9,17,18,19,20,21,22,23]. However, recommendations on meal size, macronutrient composition, and specific dietary patterns remain based mainly on physiological plausibility, general nutrition evidence, or extrapolation from related fields rather than on direct heatwave-specific intervention studies. The concept of “eating light” should therefore not be interpreted as reducing energy, protein, or micronutrient intake, especially in vulnerable groups.

The Mediterranean diet may provide a useful regional model because it emphasizes seasonal plant foods, water-rich fruits and vegetables, legumes, whole grains, olive oil, culinary simplicity, and minimally processed foods [9,21,22,34,35,36,49,50]. Nevertheless, Mediterranean diet adherence has not been directly demonstrated to improve heat tolerance, hydration status, or heat-related clinical outcomes. Therefore, it should be interpreted as a culturally rooted and potentially sustainable dietary pattern rather than as a proven heat-protective intervention.

From a public health perspective, nutrition and hydration should be incorporated into heat-health action plans as supportive preventive components. Effective strategies should include targeted communication, clinical risk assessment, medication-aware hydration advice, institutional hydration and menu protocols, food safety guidance, workplace protection, community outreach, and social support for vulnerable groups [37,38,39,40,41,42,43,44,45,46,47,48]. These actions should be integrated with environmental cooling, urban planning, occupational protection, clinical care, emergency response, and equity-oriented policies.

Overall, the available evidence supports the biological plausibility and public health relevance of hydration and dietary strategies during high-temperature periods, but direct heatwave-specific intervention studies remain limited. Future research should clarify which hydration protocols, dietary strategies, electrolyte approaches, food safety interventions, and population-specific measures are most effective, for whom, and under which environmental and clinical conditions. In a warming climate, integrating evidence-informed nutrition and hydration guidance into climate-resilient public health policies may help protect vulnerable populations and support healthier and more sustainable food systems [52].

## Figures and Tables

**Table 1:** Nature and directness of evidence supporting major nutrition and hydration recommendations during heat exposure.

Recommendation/Topic	Main Population or Setting	Nature and Directness of Evidence	Interpretation for Heat-Health Guidance
Regular water intake and dehydration prevention	General population; older adults; vulnerable groups	Public-health guidance; hydration physiology; clinical dehydration literature	Core recommendation during hot weather. Advice should be adapted to age, thirst perception, clinical status, medication use, and access to safe water [1,6,7,8,10,11,12,13]
Electrolyte replacement	Outdoor workers; athletes; high-sweat populations	Sports and occupational heat evidence; extrapolated for the general population	May be useful during prolonged or intense sweating but should not be promoted as a universal heatwave recommendation [3,14,15,16]
Avoidance of overhydration and hyponatremia	Athletes; outdoor workers; prolonged exertion in heat	Direct evidence mainly from exertional settings	Hydration advice should avoid both underhydration and excessive water intake, especially during prolonged activity in the heat [14]
Avoidance of alcohol and moderation of sugar-sweetened beverages	General population; cardiometabolic-risk groups	Public-health guidance; physiological rationale; general nutrition evidence	Water should remain the first-line beverage. Alcohol and sugar-sweetened beverages should not be promoted for hydration during heatwaves [1,7,8]
Water-rich foods	General population; older adults; children	Dietary reference values; general nutrition evidence; indirect heat evidence	Fruits, vegetables, soups, milk, yogurt, and other high-moisture foods may support total water intake and diet quality, but should not replace safe drinking water [6,10,17]
Smaller, digestible meals	General population; older adults; frail individuals	Diet-induced thermogenesis literature; physiological plausibility	Plausible strategy for comfort and tolerance during hot weather, but not directly proven to reduce heat-related morbidity [18,19]
Protein and energy adequacy	Older adults; frail individuals; workers; athletes; pregnant women	General nutrition and clinical evidence; indirect heat evidence	Advice to “eat light” should not compromise energy, protein, or micronutrient intake in vulnerable groups [11,12,13,20]
Mediterranean dietary pattern	Mediterranean/European populations	General nutrition and sustainability evidence; indirect heat evidence	Useful regional model of seasonal, plant-forward, minimally processed eating, but not a proven heat-protective intervention [9,21,22]
Micronutrients, polyphenols, and bioactive compounds	General population; selected clinical or performance contexts	Mechanistic plausibility; general nutrition evidence	Whole-food diet quality should be prioritized. Broad supplementation for heatwave protection is not currently supported [20]
Food safety during heatwaves	Households; institutions; vulnerable groups	Public health and food-safety guidance	Should be integrated into heat-health advice because high temperatures increase food spoilage and microbial risk [23]
Tailored guidance for vulnerable groups	Older adults; children; pregnant women; chronic disease; medication use; socially disadvantaged groups	Heat-health guidance; clinical/epidemiological evidence; variable directness	Recommendations should be population-specific and clinically contextualized; generic advice may be insufficient or inappropriate [1,2,5,24,25,26,27,28,29]

Note: This evidence classification is narrative and descriptive and does not represent a formal GRADE assessment. Directness refers to whether the evidence specifically addresses human heat exposure, hydration status, thermoregulation, heat-related illness, or heatwave-related outcomes. Evidence extrapolated from sports, occupational, mechanistic, or general nutrition studies was interpreted cautiously.

## Data Availability

No new data were created or analyzed in this study. Data sharing is not applicable to this article.

## Supplementary Materials

The following supporting information can be downloaded at https://www.mdpi.com/article/10.3390/nu18152502/s1. Table S1: Database-specific search strategies used for the structured narrative review.

## Abbreviations

The following abbreviations are used in this manuscript:

EFSA	European Food Safety Authority
IPCC	Intergovernmental Panel on Climate Change
WHO	World Health Organization
