# Supplementary material for: Nutrition and Hydration Strategies for Heatwave Adaptation: A Narrative Review
Source: Nutrients. 2026 Aug 3;18(15):2502. doi: 10.3390/nu18152502 (PMC13468048; doi:10.3390/nu18152502)
Supplement: Supplementary file 1 [file nutrients-18-02502-s001.zip › nutrients-4458614-supplementary.pdf]

Supplementary Table S1. Database-specific search strategies used for the structured narrative review.

Source	Search strategy / search approach	Limits and notes
**PubMed/MEDLINE**	("climate change" OR "extreme heat" OR heatwave* OR "heat stress" OR "high ambient temperature") AND (hydration OR dehydration OR "fluid intake" OR "water intake" OR electrolyte* OR sodium OR "fluid replacement")	Search fields: title/abstract. Publications in English or Italian were prioritized. The search focused mainly on publications from 2010 to 30 June 2026, while older landmark papers were retained when relevant.
**PubMed/MEDLINE**	("heat exposure" OR "heat stress" OR heatwave*) AND (nutrition OR diet OR "dietary intake" OR "meal composition" OR "water-rich foods" OR "food intake" OR "food safety")	Used to identify literature on dietary intake, food safety, and nutritional recommendations during high-temperature periods.
**PubMed/MEDLINE**	("heat stress" OR "heat exposure" OR heatwave*) AND ("older adults" OR elderly OR children OR pregnancy OR pregnant OR "chronic disease" OR "kidney disease" OR "heart failure" OR "vulnerable populations")	Used to identify evidence on vulnerable groups and population-specific heat-health risks.
**PubMed/MEDLINE**	("occupational heat exposure" OR "occupational heat stress" OR "exercise heat stress" OR "sports nutrition") AND (hydration OR dehydration OR electrolytes OR sodium OR "fluid replacement")	Used to identify evidence from occupational and sports settings. These studies were interpreted as extrapolated evidence when applied to the general population during heatwaves.
**PubMed/MEDLINE**	("Mediterranean diet" OR "plant-forward diet" OR "sustainable diet") AND (sustainability OR "climate change" OR seasonality OR "environmental sustainability")	Used to identify literature on Mediterranean dietary patterns, seasonality, and sustainability. These studies were interpreted as general nutrition or sustainability evidence, not as direct heatwave-specific evidence.
**Scopus**	TITLE-ABS-KEY ("climate change" OR "extreme heat" OR heatwave* OR "heat stress" OR "high ambient temperature") AND TITLE-ABS-KEY (hydration OR dehydration OR "fluid intake" OR "water intake" OR electrolyte* OR nutrition OR diet OR "food safety" OR "vulnerable populations")	Publications in English or Italian were prioritized. Search focused mainly on 2010–30 June 2026.
**Scopus**	TITLE-ABS-KEY ("occupational heat exposure" OR "exercise heat stress" OR "heat strain") AND TITLE-ABS-KEY (hydration OR "fluid replacement" OR sodium OR electrolytes OR dehydration)	Used to identify occupational, exercise, and sports nutrition evidence relevant to fluid and electrolyte replacement.
**Scopus**	TITLE-ABS-KEY ("Mediterranean diet" AND sustainability) OR TITLE-ABS-KEY ("Mediterranean diet" AND seasonality) OR TITLE-ABS-KEY ("sustainable healthy diets")	Used to identify literature on Mediterranean diet and sustainable food systems.
**Web of Science**	TS=("climate change" OR "extreme heat" OR heatwave* OR "heat stress") AND TS=(hydration OR dehydration OR "fluid intake" OR electrolyte* OR nutrition OR diet OR "food safety")	Used as a complementary search to identify climate-health, hydration, and nutrition literature.
**Web of Science**	TS=("heat exposure" OR "heat stress" OR heatwave*) AND TS=("older adults" OR children OR pregnancy OR "vulnerable populations" OR "chronic disease")	Used to identify evidence on vulnerable groups during heat exposure.
**Web of Science**	TS=("Mediterranean diet" OR "sustainable diet") AND TS=(sustainability OR seasonality OR "climate change" OR "food systems")	Used to identify literature on Mediterranean dietary patterns, sustainability, and climate-related food-system issues.
**Google Scholar**	Search phrases included: "heatwaves hydration nutrition", "extreme heat dehydration public health guidance", "heat health action plans hydration", "heatwaves food safety", "Mediterranean diet sustainability heatwaves", "nutrition heat stress vulnerable populations"	Used mainly to identify additional reviews, guidance documents, institutional reports, and references not retrieved through database searches. Screening was purposive and focused on relevance to the review objectives.
**Institutional websites**	Websites of international and national organizations were searched using combinations of: heat and health; heatwaves; hydration; dehydration; food safety; vulnerable populations; heat-health action plans; climate change and health; sustainable healthy diets	Sources included public health and scientific organizations relevant to heat-health prevention, climate change, hydration, food safety, and sustainable diets. Institutional documents were used primarily as public-health guidance or expert-consensus evidence.
**Citation searching**	Reference lists of highly relevant reviews, guidance documents, and landmark publications were screened manually to identify additional sources.	Used to capture key references on heat-health guidance, hydration physiology, vulnerable populations, occupational heat exposure, and sustainable diets.

Note: The search was conducted as part of a structured narrative review and was exploratory, iterative, and purposive rather than exhaustive. Searches were completed on 30 June 2026. Publications in English and Italian were prioritized, with emphasis on studies from 2010 onward; older landmark papers were included when relevant to physiological mechanisms, hydration requirements, or heat-health risks. Complete database-level record counts were not prospectively recorded, and no PRISMA-style flow diagram was generated.
